# Molecular classification of endometrial cancer: preliminary experience from a single Portuguese academic center

**DOI:** 10.3389/pore.2024.1611835

**Published:** 2024-08-16

**Authors:** João Casanova, Ana G. da Costa, Ana Pestana Lopes, Ana Catarino, Mónica Nave, Ana Carla Sousa, Jorge Lima

**Affiliations:** ^1^ Gynecologic Oncology Unit, Obstetrics and Gynecology Service, Department of Surgery, Hospital da Luz Lisboa, Lisbon, Portugal; ^2^ Gynecologic Oncology Unit, Department of Pathology, Hospital da Luz Lisboa, Lisbon, Portugal; ^3^ Gynecologic Oncology Unit, Department of Oncology, Hospital da Luz Lisboa, Lisbon, Portugal; ^4^ GenoMed—Diagnósticos de Medicina Molecular, Lisbon, Portugal; ^5^ Obstetrics and Gynecology Service, Department of Surgery, Hospital da Luz Lisboa, Lisbon, Portugal; ^6^ Comprehensive Health Research Center (CHRC), NOVA Medical School|Faculdade de Ciências Médicas, NMS|FCM, Universidade Nova De Lisboa, Lisbon, Portugal

**Keywords:** endometrial cancer, molecular subtypes, molecular profiling, POLE mutation, DNA sequencing

## Abstract

**Background:**

Since the seminal publication of the TCGA consortium in 2013, the molecular classification of endometrial cancer has been widely accepted as a new and powerful tool to better understand the natural history of this malignancy. Adoption of routine molecular classification around the world has been limited. We sought to demonstrate our initial experience in incorporating the four molecular subtypes for endometrioid carcinomas.

**Methods:**

This was a retrospective analysis at a single center in Portugal. Molecular classification was determined using immunohistochemical staining for MMR and p53 and *Sanger Sequencing* to determine *POLE* mutation status as per published PROMISE method. Descriptive statistics were reported.

**Results:**

20 patients with endometrioid histology were included. Median age of the cohort was 64 years (range 45–76). Median Body Mass Index (kg/m^2^) was 29.81 (range 21.3–43.1). In terms of tumor grading, 16 (80%) of the endometrial carcinomas of the cohort were low-grade (either grade 1 or grade 2). 16 (80%) of the cases were FIGO stage I. Regarding the molecular classification the tumors were classified as: MMRd [n = 6 (30%)]; p53 abn [n = 2 (10%)]; NSMP (n = 10 (50%)), *POLE* ultramut [n = 2 (10%)].

**Conclusion:**

Despite the small sample size, we were able to show that molecular classification is feasible. To our knowledge this is the first cohort of endometroid endometrial carcinomas fully characterized according to the TCGA classification in Portugal, from one single center.

## Introduction

As obesity continues to increase all over the world, so is the incidence of endometrial cancer (EC). Besides obesity playing a major role in the development of endometrial cancer, other risk factors such as diabetes, polycystic ovarian syndrome, estrogen-secreting tumors, unopposed estrogen replacing hormone therapy and tamoxifen use, they all predispose women to endometrial cancer [1]. Historically endometrial carcinomas have been classified using a two-tier classification, type I usually comprising estrogen related tumors and type II non-estrogen/hormone related tumors. Pathologically, type I comprises endometrioid histology and affects roughly 80% of the patients; and type II comprises non-endometrioid histology and it affects the remaining 20% of patients [2]. As previously noted, hormonal factors, namely estrogen excess plays a major role in the etiopathogenesis of endometrioid-type endometrial carcinomas [1]. Focusing more on this subtype, according to the International Federation of Gynecology and Obstetrics (FIGO), endometrioid endometrial carcinomas are graded in a three-tier system, noting the relative proportion of glandular and solid-tumor components [3]. Grade 1 and 2 endometrioid endometrial carcinomas are usually called “low-grade” tumors and are associated with a favorable prognosis. Grade 3 tumors are also called “high-grade” tumors and are usually associated with and intermediate/poor prognosis [1].

In 2013, The Cancer Genome Atlas (TCGA) research network performed and integrated genomic and transcriptomic analysis of 373 endometrial cancers [4]. These findings translated into a proposed molecular classification for EC, comprising four molecular subtypes: DNA Polymerase epsilon (*POLEmut*) ultramutated, microsatellite instability (MSI) hypermutated, copy number high and copy number low [4]. To simplify all these new findings the Proactive Molecular Risk Classifier for Endometrial Cancer (ProMisE) guidelines were established and distinguished the following subtypes of EC: mismatch repair deficiency (MMRd), p53 abnormal (p53abn), non-specified molecular profile (NSMP) and *POLE* ultramutated [5]. These new guidelines are currently more widespread, as the complex and expensive genetic sequencing used by the TCGA consortium can be replaced by immunohistochemistry surrogates to determine most of these profiles [6]. Currently only the *POLE* status needs to be determined by DNA sequencing. For the five most common *POLE* mutations (P286R, V411L, S297F, A456P and S459F), pathogenicity (causing tumor ultramutation) has been confirmed [7].

The firm establishment of these new molecular categories of EC has had a significant impact and interest in a more tailored adjuvant therapy, particularly since the PORTEC-3 trial showed a strong prognostic value of the molecular classification in high risk EC [8]. Interestingly, robust data in the literature has been consistent in demonstrating that *POLE* ultramutated tumors have an incredible favorable prognosis [1, 4, 8–10]. More recently, the new FIGO guidelines for EC staging include the new molecular profiling highlighting that this is the new standard of care [9].

Despite all data available, to the authors knowledge, *POLE* testing has not been widely used in Portugal. The authors would argue that this is mainly due to the absence of available surrogates for *POLE* sequencing. Other factor would be related to health cost, namely DNA sequencing.

**FIGURE 1:**
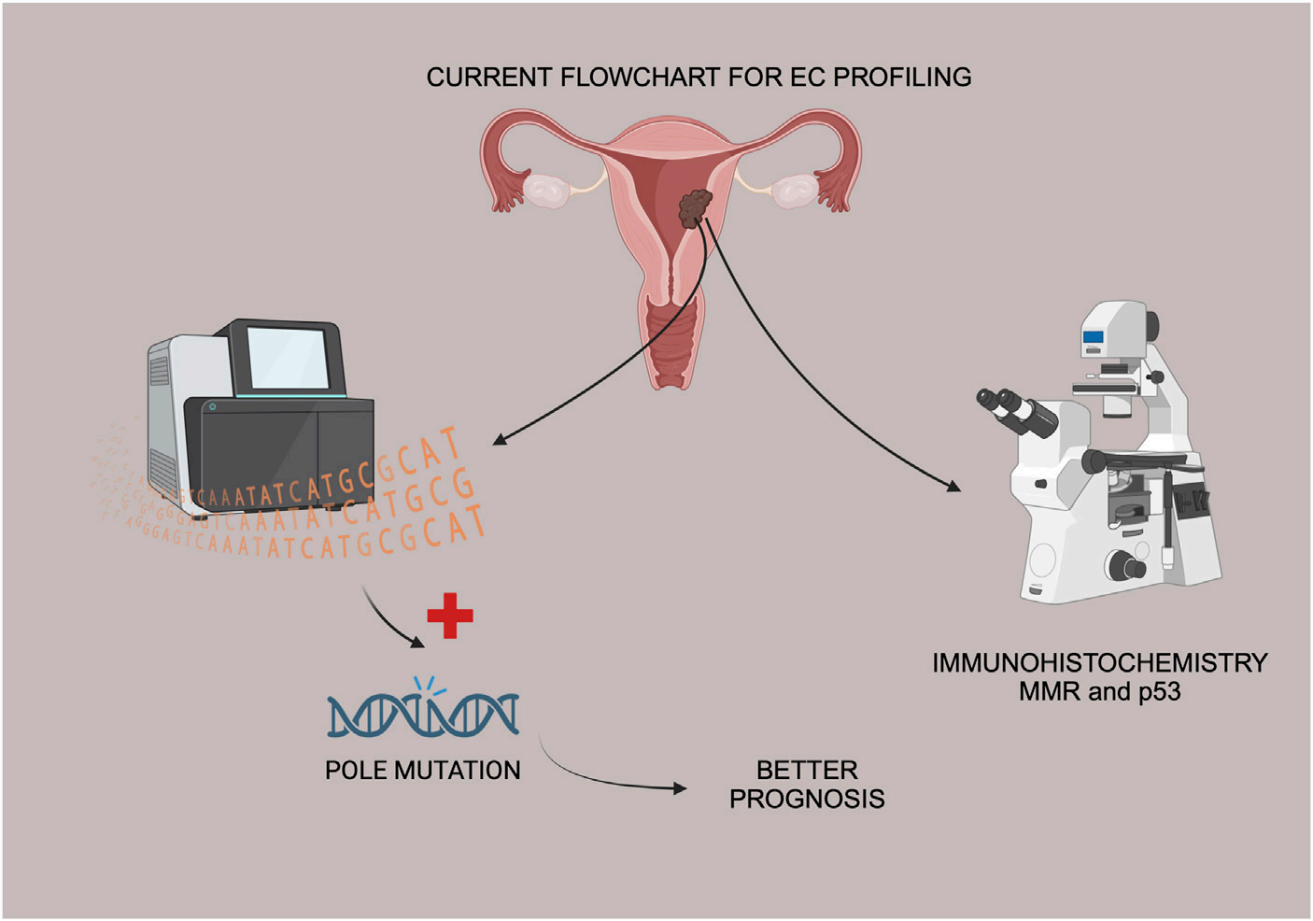
Flowchart of EC tissue samples, IHC and Sanger sequencing for *POLE* mutation status.

According to the landmark paper by the TCGA group, *POLE* ultramutated endometrial carcinomas are a subgroup of endometrioid EC, representing roughly 10% of the endometrioid histology [4]. Data from the literature supports this finding and this was why the authors decided to focus this preliminary analysis only in endometrioid EC (Figure 1).

## Materials and methods

### Cohort

This retrospective study was approved by the local Ethics Committee. Formalin-fixed paraffin-embedded tissue blocks of 20 patients diagnosed between January of 2022 and February of 2024 with endometrioid EC at a tertiary/academic hospital were retrieved. Informed consent was obtained from all patients. The inclusion criterion was endometrioid histology. Biopsy samples were prioritized due to more potential tumor cells (either from hysteroscopy, curettage and Pipelle biopsy). If these tissue samples were not available, tissue from the surgical specimen containing the primary tumor was used.

### DNA sequencing

The detection of POLE somatic mutations was performed at an external certified laboratory using Sanger Sequencing. Mutation screening of exons 9, 11, 13, and 14 of the *POLE* gene (12q24.33, OMIM#174762, NM_006231.3, and LRG_789t1) was conducted in samples with tumor content higher than 20% and subsequently analysed by Sanger sequencing. Exonic regions of interest were amplified by polymerase-chain-reaction (PCR) with flanking intronic primers and sequenced by an automatic sequencer (ABI Prism 3100-Avant Capillary Array, 36 cm, Applied Biosystems). Data was evaluated using the DNA Sequencing Analysis Software 6™ Version 6.0 (Applied Biosystems).

### Immunohistochemistry

**FIGURE 2:**
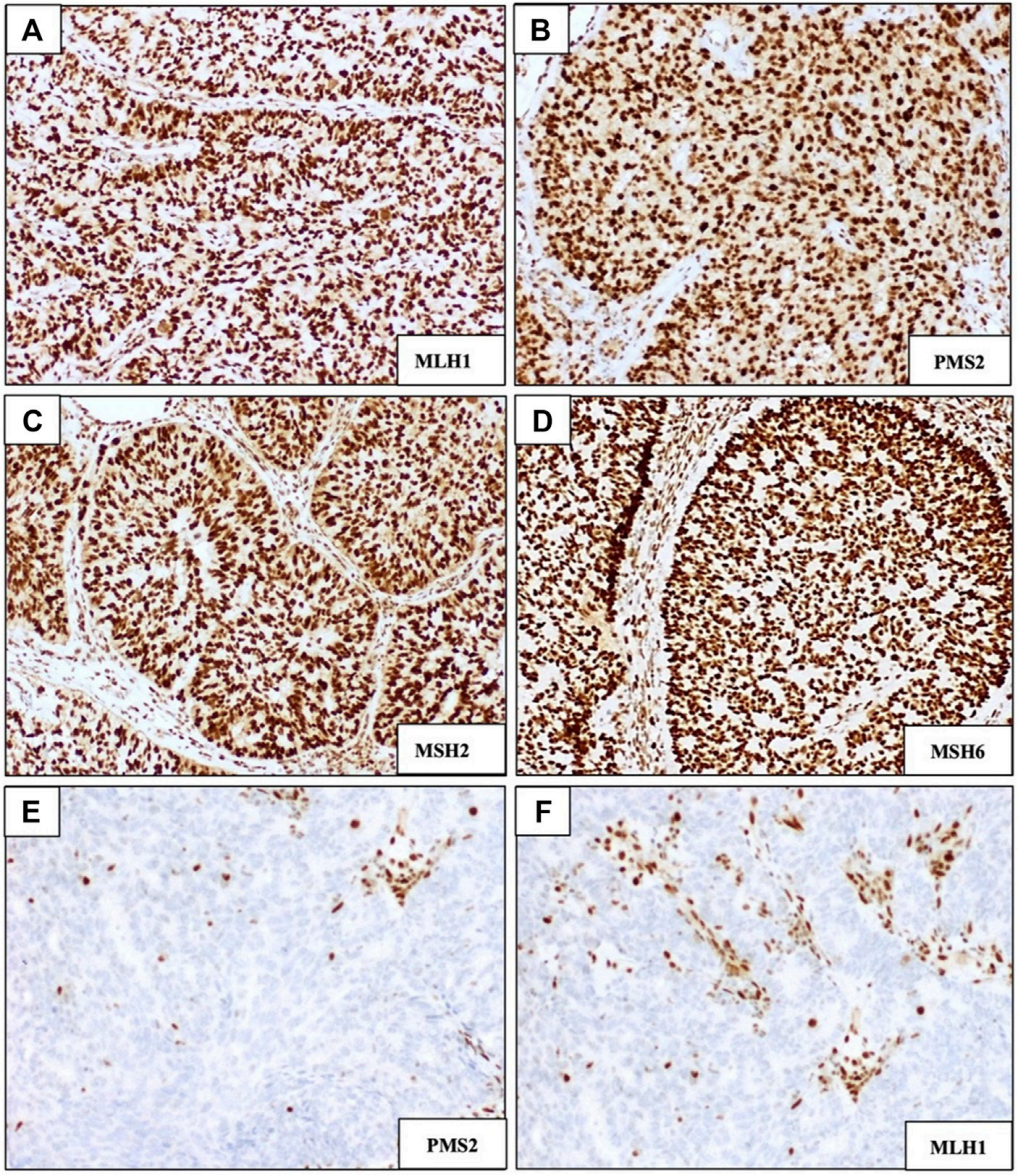
Immunohistochemical staining of DNA mismatch repair (MMR) markers MLH1, PMS2, MSH2, and MSH6. **(A–D)** Examples of DNA MMR-proficient staining patterns showing intact/retained nuclear staining. **(E,F)** Examples of MMR-deficient staining patterns showing loss of expression of PMS2 and MLH1, respectively; (magnification ×100).

MMR status was investigated by immunohistochemistry (IHC), evaluating the presence or absence of four proteins involved in the mismatch repair pathway: MLH1, PMS2, MSH2, and MSH6. p53 abnormalities were also analysed using IHC. A representative slide of each sample was selected, and then three-micron-thick serial paraffin sections were processed by IHC using an automated immunostainer (Ventana BenchMark Ultra). Each IHC marker was examined under light microscopy by two pathologists. Regarding to MMR enzyme immunohistochemistry, cases were scored as “retained” if uniform intact nuclear staining for the protein was observed, whereas MMRd tumors were characterized by a complete absence of intact nuclear staining (Figure 2). The specifications of the antibodies used for MSI staining were as follow: Ventana anti-MSH2 (G219-1129), mouse monoclonal primary antibody, 5 mL (∼20 μg/mL); Ventana anti-PMS2 (A16-4), mouse monoclonal antibody, 5 mL (∼1 μg/mL); Ventana anti-MLH1 (M1) mouse monoclonal primary antibody, 5 mL (∼1 μg/mL); Ventana anti-MSH6 (SP93) rabbit monoclonal primary antibody, 5 mL (∼1 μg/mL).

**FIGURE 3:**
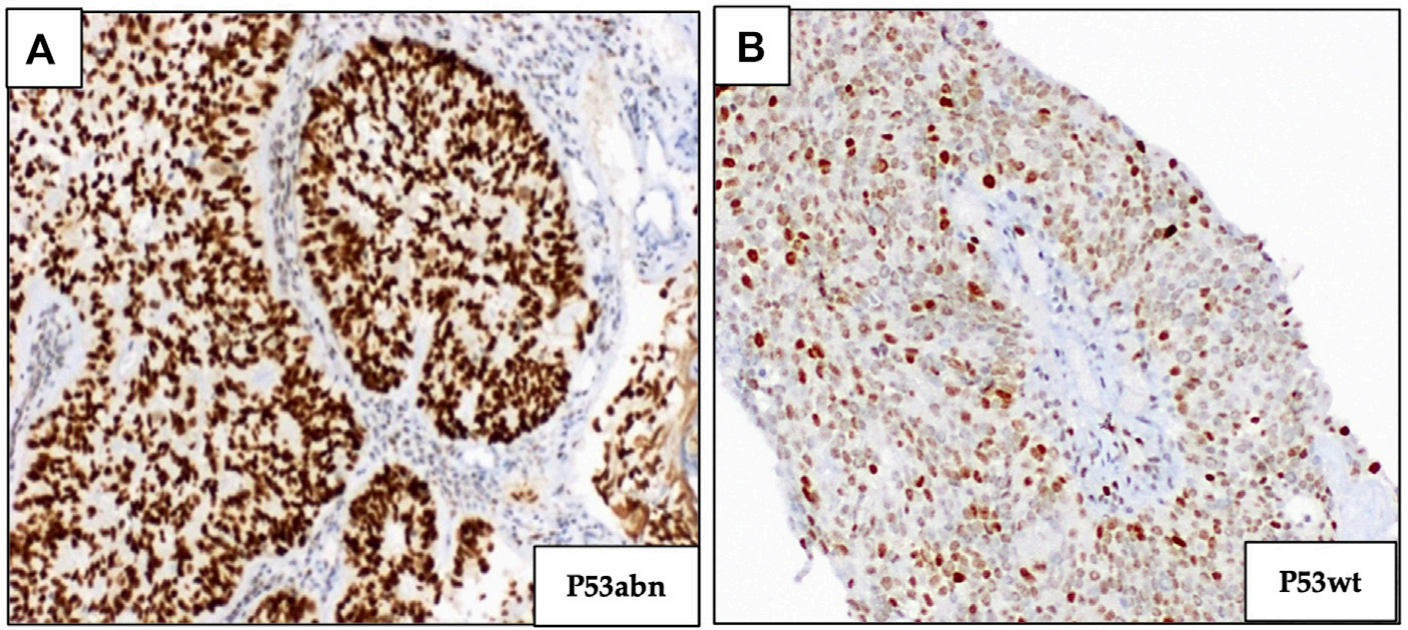
Immunohistochemical staining of p53 marker. **(A)** p53 overexpression (abnormal p53). **(B)** Normal expression of p53 (p53 wild type); (magnification ×100).

Abnormal p53 (p53abn) staining was recognized either by overexpression (diffuse strong staining of >75% tumor cell nuclei) or a complete absence of staining of tumor cell nuclei, in the presence of an intact internal control. p53 wild-type (p53wt) tumors were characterized by scattered nuclear staining with a mixture of negative, weak, and strong staining of tumor cell nuclei (Figure 3). Ventana CONFIRM anti p53 (DO-7) primary antibody, 5 mL (∼0.5 μg/mL) was used for p53 staining.

## Results

DNA sequencing to determine *POLE* status was successful in all cases. IHC was performed in all samples. The clinicopathologic characteristics of the cohort are listed in Table 1. The 2009 FIGO Staging System of endometrial cancer was used.

**TABLE 1:** Clinicopathologic characteristics of the EC cohort.

Clinicopathologic characteristics	N = 20^a^
Age (years) Median (range)	64 (45–76)
BMI (kg/m^2^) Mean (range)	29.57 (21.3–43.15)
Tumor Grade	
Low Grade (G1&2)	16 (80%)
High Grade (G3)	4 (20%)
Lymphovascular Invasion	
Yes	4 (20%)
No	15 (75%)
FIGO Stage^b^	
IA	12 (63%)
IB	4 (21%)
II	0
III	2 (10%)
IV	1 (5%)
Adjuvant Therapy	
None	11 (58%)
Radiotherapy	4 (21%)
Chemotherapy	1 (5%)
Chemo-Radiation Therapy	3 (16%)
SLN	
Yes	16 (84%)
No	3 (16%)
MIS	
Yes	18 (95%)
No	1 (5%)

SLN, Sentinel Lymph Node Dissection; MIS, Minimally invasive Surgery (robotic surgery or laparoscopic surgery).

^a^ One patient pursued treatment at other medical institution.

^b^ 2009 FIGO, staging system of Endometrial Cancer.

Afterwards, the EC samples were classified into one of the four molecular subgroups:

• MMR*d* – 6 (30%)
• p53*abn* – 2 (10%)
• NSMP – 10 (50%)
• POLE ultramutated – 2 (10%)

Regarding the specific location of the POLE mutations, one case had the mutation (c1366G>C p (Ala456Pro) in exon 14. The other patient with a POLE ultramutated tumor had the mutation (c.857C>G p.(Pro286Arg) in exon 9.

One *POLE* ultramutated tumor was identified in a patient included in the multiple classifiers group (this patient had a tumor with both p53*abn* and MMR*d*). The other POLE ultramutated tumor was an early stage (FIGO IB) low-grade tumor. Regarding MMRd tumors, four cases had MLH1 and PSM2 deficiency, one case had MSH6 and MSH2 deficiency and one case had MSH6 and PSM2 deficiency. Both p53*abn* tumors were high-grade tumors, but interestingly both were FIGO Stage I. Regarding the six MMR*d* tumors, five tumors were low-grade (either grade 1 or grade 2) and one was high-grade. Among the MMR*d* tumors, four cases presented as FIGO Stage I, one as FIGO Stage IIIC1 and another one as FIGO stage IVB. Regarding the surgical approach, the majority of patients (95%) underwent MIS and one patient underwent a primary cytoreductive surgery, via laparotomy. SLN biopsy was performed in 84% of the cases. Table 2 shows every patient classified according to the molecular subtype. FIGO stage, tumor grading and adjuvant therapy are also described.

**TABLE 2:** Molecular classification of the EC cohort.

Patient	Grade	FIGO Stage	Subtype	Adjuvant therapy
A	2	IA	NSMP	None
B	2	IIIC1	NSMP	Chemo-Radiation
C	1	IA	MMR*d*	None
D	3	IVB	MMR*d*	Chemotherapy
E	2	IIIC1	MMR*d*	Chemo-Radiation
F	1	IA	NSMP	None
G	3	IA	p53*abn*	Radiation
H	1	IA	NSMP	None
I	3	IB	MMR*d*	Chemo-Radiation
J	3	IA	p53*abn*	Radiation
K	1	IA	NSMP	None
L	1	IA	MMR*d*	None
M	1	IA	*POLEult*	None
N	1	IA	MMR*d*	None
O	1	IA	NSMP	None
P	1	IA	NSMP	None
Q	2	N/A	NSMP	N/A
R	1	IB	NSMP	Radiation
S	2	IB	NSMP	Radiation
T	2	IB	*POLEult*	None

N/A, data not available.

## Discussion

As previously discussed, EC is one of the most common gynecologic malignancies and its incidence is rising [11]. The advances catalyzed by the TCGA consortium, have now set molecular profiling of endometrial cancer as an extremely useful tool, with major impacts in refining adjuvant therapy and with prognostic implications. Of note, the *POLE* ultramutated subgroup of EC seems to carry an excellent prognosis [12–14]. Regarding MSI EC (or MMR*d*) data in the literature found that these tumors were more likely to present with poor prognostic factors, such as higher grading and frequent lympho-vascular space invasion [15]. Interestingly, the clinical outcome of these patients seems to be more on the favorable side [15–18]. Regarding p53*abn* EC, they carry the worst prognosis and most likely benefit from aggressive adjuvant therapy [19]. Lastly the NSMP endometrial carcinomas have been mostly classified as intermediate risk, but further refining is necessary to have a more tailored approach to these tumors [20]. To our knowledge, this is the first cohort of Portuguese patients, from one single center, with endometrioid EC to be fully classified according to the molecular profile. In all twenty patients of the cohort, the IHC studies were performed, and *POLE* sequencing was successful. As expected, most of the patients had low grade and early-stage tumors. Two *POLE* ultramutated tumors were identified in our cohort. According to the literature, *POLE* ultramutated tumors are approximately 10% of all endometrioid EC so the authors expect that as the sample size will grow, the corresponding *POLE* mutations will be identified. We would also mention that one of the POLE mutated patient was not submitted to adjuvant brachytherapy. This decision was taken after discussion in our tumor board meeting and there is robust data supporting this, including the new FIGO staging system for EC [9]. The authors would like to highlight that both p53*abn* EC were early stage (FIGO Stage IA), and underwent adjuvant therapy, after discussion in a multidisciplinary decision management meeting. In Portugal, due to limited resources specifically in genetic sequencing facilities, to our knowledge, the “new” molecular EC classification has not been fully embraced by most healthcare institutions, either in the public and private sector. As previously mentioned, the molecular profiling of EC has major prognostic implications, and as data in the literature matures, significant changes in the management of EC are underway [9]. For instance, PORTEC4a, will be addressing the risk of vaginal recurrence in women with high-intermediate risk EC treated after surgery with molecular-integrated risk profile-based recommendations (observation, vaginal brachytherapy or external pelvic beam radiotherapy versus standard adjuvant vaginal brachytherapy) [21]. Recently, the ESGO/ESTRO/ESP published in 2021, encourage molecular profiling of all EC, especially high-grade tumors [22]. Regarding high-grade tumors, specifically high-grade EC, having a full molecular-integrated risk profile can be extremely useful in “de-escalating” adjuvant therapy if certain conditions apply. As an example, studies have shown that *POLE* mutations are common in high-grade endometrioid EC, and that these tumors, G3 *POLEmut* have favorable oncologic outcomes and possibly a more conservative adjuvant approach can be recommended in a near future [23–27]. Our group recently published a systematic review and meta-analysis supporting these findings [10]. Bearing all the above, our objective with this preliminary study was to investigate if it would be possible with appropriate funding, to cooperate with a genetic testing certified laboratory and address the lack of data in Portugal regarding the molecular profile of endometrioid EC.

As for the main limitations of this study, the small number of patients enrolled is the most important one. However, being this a preliminary experience, we feel that it would be important to “start small and then go big” as it was necessary to establish new protocols with a certified laboratory regarding *POLE* sequencing and internally validate the data, before setting molecular profiling of EC as the standard of care in our institution. Despite that the literature has robust data regarding the importance of having a full molecular profile for EC, the authors feel that having data of a very small cohort of Portuguese patients can help to disseminate the clinical relevance of this fact. Other limitation that the authors would like to point out is related with the necessity of sequencing the *POLE* in all EC. The group that developed The Proactive Molecular Risk Classifier for EC (ProMiSe) has published several studies, mentioning that POLE sequencing may be omitted in low-risk EC, with low risk histologies [6, 28]. To this fact the we would like to point out that routine IHC (p53, MMR) was already being routinely performed at our institution, however, all genetic sequencing had to be done at an outside laboratory so the authors felt that it would be important to establish routines, and again to internally validate the data. We also expect that by leading this initiative in our country more collaboration will be established and we can move forward in offering a comprehensive histologic and molecular profile to all patients with EC.

## Data Availability

The original contributions presented in the study are included in the article/supplementary material, further inquiries can be directed to the corresponding author.
